# The potential impact of lipopolysaccharides (LPS) interactions on light-activated ion channels and pumps

**DOI:** 10.17912/micropub.biology.001463

**Published:** 2025-03-12

**Authors:** Youngwoo Kim, Jiwoo Kim, Victoria Spedding, Robin Cooper

**Affiliations:** 1 Biology, University of Kentucky, Lexington, Kentucky, United States; 2 Life sciences, The Gatton Academy, Western Kentucky University, Bowling Green, Kentucky, United States; 3 Life sciences, Model Laboratory School, Eastern Kentucky University, Richmond, Kentucky, United States; 4 University of Massachusetts Chan Medical School at Baystate Medical Center, Springfield, Massachusetts, United States

## Abstract

Optogenetics, the expression of light-activated ion channels and pumps, has the potential in therapeutic treatments for various pathologies. Thus, it is of interest to investigate pharmacological agents, immunological responses, and disease states like bacterial septicemia to learn if light-activated ion channels and pumps are compromised. Bacterial septicemia may impede the function of light-activated channels due to direct actions of lipopolysaccharides. This study explored the effects of activating light-sensitive channels (channelrhodopsin and halorhodopsin) before, during, and after exposure of lipopolysaccharides. Lipopolysaccharide itself did not directly impede the action of LPS but varied because of the effect on ion gradients by changes in membrane potential by lipopolysaccharides.

**Figure 1. The effect of lipopolysaccharide (LPS) on the responses of channelrhodopsin-2 (ChR2-XXL) and halorhodopsin (eNpHR) activation and vice versa:**
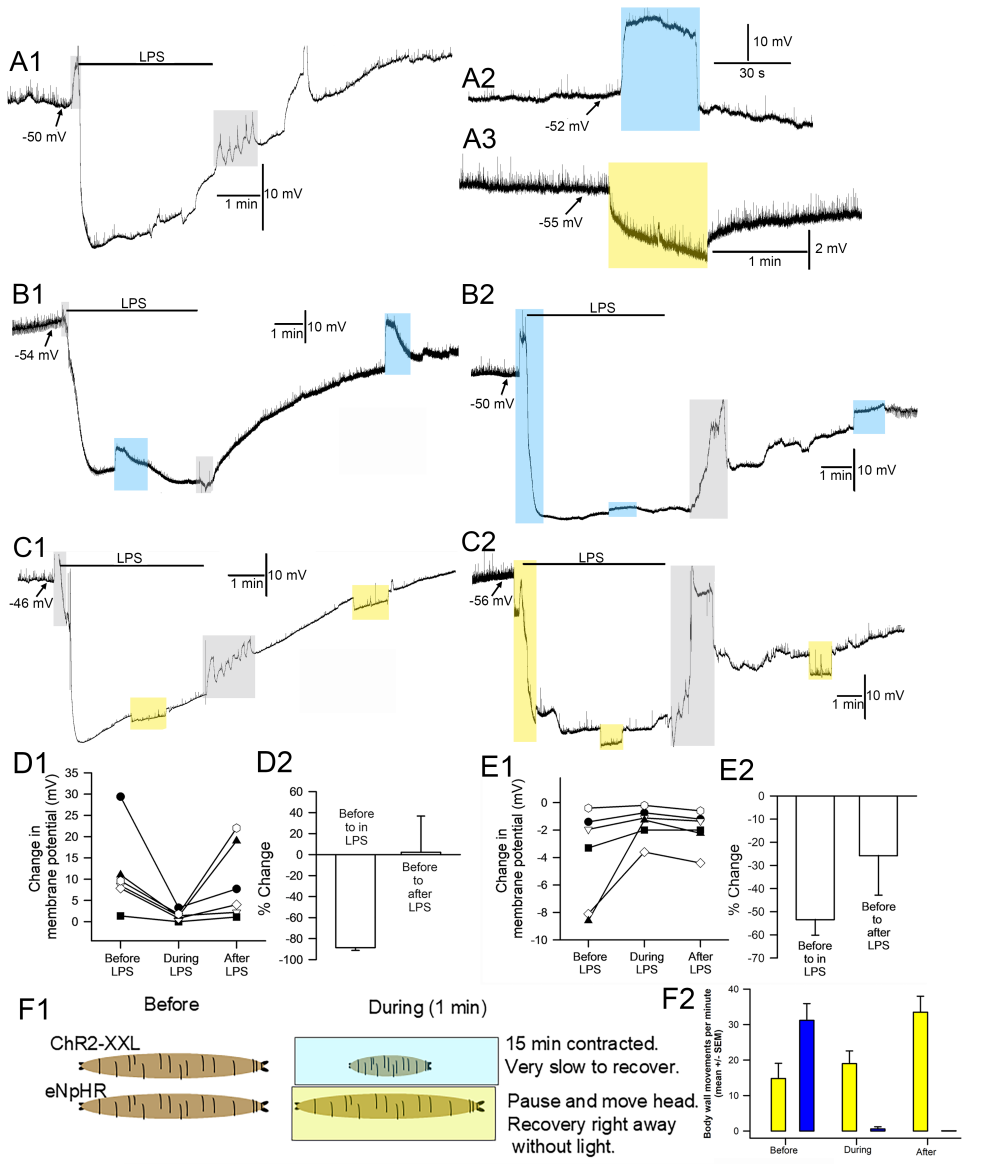
(A1) The effect on membrane potential by LPS from *Serratia marcescens* (500 µg/ml). (A2) The effect of blue LED on membrane potential for muscle 6 seg 2 of larvae expressing ChR2XXL. (A3) The effect of yellow LED on membrane potential for muscle 6 seg 2 of larvae expressing eNpHR. (B1) The effect on membrane potential by LPS and while exposed to LPS the action of activating ChR2XXL as well as after LPS is removed. (B2) First activating ChR2XXL and while activated exposure to LPS. Followed by ChR2XXL during LPS exposure as well as after LPS is removed. (C1 & C2) The same paradigms as in B1 and B2 but with activating eNpHR. (D1) The change in membrane potential (i.e. depolarization) during blue light exposure for each preparation before, during and after LPS exposure (p<0.05; paired t-test). (D2) The percent change in membrane potential before to during LPS exposure and before to after LPS exposure. (E1) The change in membrane potential (i.e. hyperpolarization) during yellow light exposure for each preparation before, during and after LPS exposure (p<0.05; paired t-test). (E2) The percent change in membrane potential before to during LPS exposure and before to after LPS exposure. (F1) The generalized effects of intact crawling larvae observed in IR lighting while activating either ChR2XXL or eNpHR. (F2) The body wall movements of larvae before, during and after light exposure. The larvae expressing eNpHR still were crawling during the light exposure and even speed up after the light was turned off (p<0.05; paired t-test, yellow bars). The larvae expressing ChR2XXL stopped moving and remained contracted for up to 15 minutes before they even started to try to move (p<0.05, paired t- test, blue bars). The gray boxes are times when the bathing media was exchanged, which results in electrical disturbances. The blue boxes are when ChR2XXL is activated and yellow boxes when eNpHR is activated.

## Description

The use of light-activated ion channels and pumps have revolutionized life sciences in being able to have these proteins selectively expressed in tissues and cell subtypes. The two commonly used light-sensitive proteins for research are channelrhodopsin-2 (ChR2) and halorhodopsin (NpHR) (Zhang et al., 2007). ChR2 causes an influx of Ca ^2+^ and Na ^+^ (Nagel et al., 2003), while halorhodopsin pumps chloride ions into cells (Duschl et al., 1990). A number of reviews in the history of their development in research, how they function, modifications in their structure to alter sensitivity to light, and potential uses and pharmacological profiling have been published (Gradinaru et al., 2010; Joshi et al., 2020; Gilhooley et al., 2022; Spreen et al., 2024). Since the light sensitive channels are touted for many medical treatments (i.e., Parkinson’s, epilepsy, neuromuscular disorders), it is of interest to know if therapeutic pharmacological agents, immunological responses, and particular disease states, such as bacterial septicemia, may compromise or alter the function of the light-activated ion channels and pumps.

It is known that the non-selective blocker of ion channels GdCl _3_ (gadolinium chloride at 10 mM) the voltage- gated sodium channel blocker tetrodotoxin (5 μM) and a high concentration of ethanol (100–300 mM) can dampen the currents by ChR2; however, morphine (30–100 μM) can inhibit the current (Gioia et al., 2018). Specific inhibitors for the NpHR chloride pump are not established. For the light-activated proteins to be used in clinical applications, they need to be examined in various pathological conditions with exposure to various pharmacological agents and potentially in unison. In the case of disease states associated with Gram-negative bacterial septicemia, the toxin lipopolysaccharide (LPS), also known as a endotoxin, is released from the bacteria to result in immunological responses by the body. Some forms of LPS are now known to not only bind to CD14/TLR4/MD2 complex for an immune response (Park and Lee, 2013) but to likely function directly on K2P (two-pore-domain) K ^+^ channels and on Na ^+^ leak channels (NALCN) (McCubbin et al., 2024). The long outer core polysaccharide tails of LPS could potentially also have an action on the ChR2 channel and the NpHR ion pump. Thus, we examined if a high concentration of an LPS form that appears to activate K2P and NALCN channels would block the function of ChR2 and NpHR effects when activated. We expressed Channelrhodopsin-2 (ChR2XXL, highly light sensitive form) or Halorhodopsin (eNpHR; an ER export directed form of NpHR) in *Drosophila* larval body wall muscles and examined the effects before, during and after exposure to LPS from *Serratia marcescens* .

After dissections and placing the intracellular electrode, the preparations were dark adapted for 5 minutes. Two general paradigms were used. The first paradigm had the light activated channels excited for 1 minute. After conducting current for 30 seconds LPS was applied with light on for the next 30 seconds. Then after 3 minutes, or until the membrane potential showed some stability, the preparation was exposed to light again for another minute. The preparation was then flushed with fresh saline and after the potential was stable (~2 to 3 min), it was exposed to light again. The second paradigm was to first expose the preparation to LPS for 2 minutes or until the membrane potential showed some stability and then expose the preparation to light. This was followed by flushing the LPS away with fresh saline and exposing the preparation to light again. LPS exposure results in hyperpolarization followed by depolarization during exposure (A1). Before LPS exposure activating ChR2XXL with blue light produces a large depolarization (A2) while activating eNpHR produces a slight hyperpolarization (A3). While exposed to LPS the responses to activating ChR2XXL (B1) or eNpHR (C1) are dampened. Light exposure right before and during the switch to LPS exposure shows the rapid depolarization for ChR2XXL followed by the LPS induced hyperpolarization (B2). A second blue light pulse during LPS produced a dampened depolarization and stronger depolarization after LPS was removed. Before LPS exposure, activating eNpHR with yellow light produces a hyperpolarization (C2) and during LPS exposure the membrane potential hyperpolarized but during LPS exposure activating eNpHR produces a smaller amount of hyperpolarization followed by a stronger response to light after LPS was removed.

Six larvae for each joint exposure of LPS and activating ChR2XXL resulted in slightly dampened response to light while hyperpolarized by LPS but showed a recovery response after removal of LPS. (D1) The amount of depolarization during ChR2XXL activation before, during and after LPS exposure. The amount of depolarization was significantly reduced during LPS exposure (p<0.05, paired t-test, D1). The average (+/-SEM) percent change before and during LPS exposure demonstrates the reduced effect caused by a change in membrane potential from LPS (D2). The effect of activating eNpHR before, during, and after LPS exposure was due to the Cl ^-^ being pumped into the cell, but as for ChR2XXL was reduced during LPS exposure (p<0.05, paired t-test, E1). The reduction in the responses by LPS were less so for eNpHR than for ChR2XXL likely due to a smaller change induce by eNpHR on membrane potential before exposure to LPS. The opening of K2P channels resulting in reducing membrane resistance and a strong K ^+^ efflux dampened the effect of the cation influx by ChR2XXL. It is concluded that exposure to LPS before or during activating ChR2XXL or eNpHR does not inhibit their function but due to LPS altering the ionic driving gradients for the effects on light activated channels may be pronounced but not for a Cl- pump. Thus, optogenetic approaches could be used in the presences of LPS from *Serratia marcescens* ; though the effects would depend on the degree of action by LPS in modulating K2P and NALCN channels.

The effects of activating ChR2XXL or eNpHR in intact larvae with these lines using the same illuminations of blue or yellow light revealed that the larvae which were selectively expressing ChR2XXL in muscle m6 and m7 resulted in larvae being contracted for at least 15 minutes. While for eNpHR the larvae paused in crawling but were able to move and as soon as the light was off, they recovered in crawling even at a greater rate than before light exposure (F1 and F2). These results serve as proof of concept that activating or inhibiting the membrane potential in m6 and m7 muscles alters the intact behavior of larvae.

## Methods

*Drosophila melanogaster* strains were obtained from the Bloomington Drosophila Stock Center (BDSC). The first filial 1 (F1) generations were obtained by crossing virgin females of a halorhodopsin strain, (w*;P{UAS-eNpHR-YFP; (BDSC stock# 41752) or channel rhodopsin strain (y1 w1118; PBac{UAS-ChR2.XXL}VK00018; BDSC stock# 58374) with males of BG487-GAL4 (larval body wall muscles (m6 and m7 in an anteroposterior gradient in larval body wall muscles 6/7; BDSC stock# 51634)(Budnik et al., 1996; Sulkowski et al., 2014). All animals were maintained in vials partially filled with a standard cornmeal-agar-dextrose-yeast medium. After crossing lines, the larvae were raised in complete darkness. All trans-retinal (ATR; Sigma-Aldrich, St. Louis, MO, USA) was diluted in standard fly food to a final concentration of 1 mM (for ChR2 use) or 10 mM (for eNpHR use) and protected from light with aluminum foil. Early 2nd instars were cultured in the ATR containing food.

The larval dissection technique has been previously reported (Li et al., 2001) for early 3rd instars. Lighting was kept to a minimum for dissection and for placing the intracellular electrode. To monitor the transmembrane potentials of the body wall muscle (m6) of 3rd instar larvae, a sharp intracellular electrode (30 to 40 megaohm resistance) filled with 3M KCl impaled the m6 fiber in segment 2. An Axoclamp 2B (Molecular Devices, Sunnyvale, CA, USA) amplifier and 1 X LU head stage were used. A modified basal HL3 saline was used (NaCl 70 mM, KCl 5 mM, MgCl _2_ ·6H _2_ O 20 mM, NaHCO _3_ 10 mM, Trehalose 5 mM, sucrose 115 mM, BES 25 mM, and CaCl _2_ ·2H _2_ O 1 mM, pH 7.1; Stewart et al., 1994; deCastro et al., 2014). The pH of the saline was maintained at 7.2. The LPS compound that was used for assessment was Serratia marcescens (product number L6136; Sigma-Aldrich). The concentration of the LPS was 500 µg/ml in order to compare and contrast to prior studies and was made fresh daily (Cooper and Krall, 2022). The high intensity LEDs used in this study were blue light (470 nm wavelength, LED supply, LXML-PB01-0040, 500 mA) and yellow-lime light (567.5 nm wavelength, LED supply, LXML-PM02-0000, 500 mA). The 470 nm blue light was used for the Chr2XXL, and 567.5 nm yellow-lime light was used for the halorhodopsin lines, based on which wavelengths resulted in maximum activation of the light-sensitive proteins as previously defined (Govorunova et al., 2015; Zhang et al, 2007). The photon flux (number of photons per second per unit area) was measured with a LI-COR (model Li-1000 data Logger, LDL3774; LI-COR from Lincoln, NE, USA) that measured µMol s−1 m−2 per µA. In addition, the full spectrum of lights was measured with a Jazz (Ocean Optics Inc., Largo, FL, USA) to obtain a total W/m2 from 340 to 800 nm spectrum for each light source.

## Reagents

NaCl

KCl

MgCl _2_ .6H _2_ O

NaHCO _3_

L-Trehalose

sucrose

N,N-bis(2-hydroxyethyl)-2-aminoethanesulfonic acid

CaCl _2_ .2H _2_ O

trans-retinal (ATR; product # R2500)

LPS from Serratia marcescens (product number L6136).

All compounds from Sigma-Aldrich, St. Louis, MO, USA.

*Drosophila melanogaster* strains Bloomington Drosophila Stock Center (BDSC).
